# Effect of insect cuticular compounds on appressorium formation and metabolic activity in *Metarhizium anisopliae*

**DOI:** 10.3389/fmicb.2026.1842196

**Published:** 2026-06-18

**Authors:** Yang Xu, Canxia Wu, Dongxu Wang, Huaxin Cai, Jingyang Ni, Wenzhe Li, Yinghua Tong

**Affiliations:** Forestry College, Fujian Agriculture and Forestry University, Fuzhou, China

**Keywords:** appressorium, insect cuticular compounds, metabolism, *Metarhizium anisopliae*, spore germination

## Abstract

The rate of appressoria formation following conidial germination in *Metarhizium anisopliae* is closely associated with its pathogenicity. This study investigated the effects of insect cuticular compounds on the formation and metabolism in *M. anisopliae* by adding insect cuticle analogues. The results showed that amino acid compounds L-aspartic acid and glycyl-L-phenylalanine, amine-class compound L-sorbitol, aromatic compounds and fenofibric acid, and organic acid 4-(aminomethyl) benzoic acid promoted spore germination and appressorium formation (*p* ≤ 0.05). Among these, L-aspartic acid significantly promoted spore germination at all tested concentrations (*p* ≤ 0.05), but showed no significant effect on appressorium formation at low concentrations (*p* > 0.05). In contrast, high concentrations significantly enhanced appressorium formation (*p* ≤ 0.05). Moreover, the promotive effect on appressorium formation increased with concentration, and at 1 mg/mL both the spore germination rate and appressorium formation rates peaked across all time points, being significantly higher than those of the control (*p* ≤ 0.05). Both epigallocatechin gallate and glycerophosphocholine promoted spore germination and appressorium formation at low concentrations, whereas high concentrations significantly inhibited both processes (*p* ≤ 0.05). At higher concentrations, the organic acid compound mitifos inhibited conidial germination and appressorium formation (*p* ≤ 0.05). For the aromatic compound 2,6-dihydroxybenzoic acid, the inhibitory effect on conidial germination and appressorium formation increased with concentration. Analysis of the effects of L-aspartic acid on metabolism during appressorium formation in *Metarhizium anisopliae* using LC-MS metabolomics showed that the amino acid compound L-aspartic acid broadly perturbed the secondary metabolism of *M. anisopliae*. After appressorium formation, a total of 367 unique differential metabolites were identified. Prior to appressorium formation, L-aspartic acid supports the energy demands of cellular assembly and proliferation by promoting the activation of *M. anisopliae* including D-amino acid metabolism, biosynthesis of cofactors and amino acid biosynthesis, thereby strengthening stress defense and signal transduction. After appressorium formation, L-aspartic acid activates pathways in *M. anisopliae* including tryptophan metabolism, the two-component system, β-alanine metabolism and folate metabolism, providing signaling cues to drive hyphal growth and accelerating toxin production and secondary metabolite biosynthesis. Thus, L-aspartic acid provides substances and energy for spore germination, appressorium cell wall thickening and stress early warning of *M. anisopliae*, and enhances fungal defense capacity. After appressorium formation, L-aspartic acid further promotes fungal cuticle penetration, accelerates in-host colonization, and facilitates toxin synthesis to kill the host, thereby improving the infectivity of *M. anisopliae* in multiple ways. This study provides a theoretical basis for in-depth investigations into the infection mechanism of *M. anisopliae* and the mechanistic role of appressorium during infection.

## 1 Introduction

The genus *Metarhizium* is a widely used pathogenic fungus that can persist in insects, ticks, nematodes, as well as soil (Schrank and Vainstein, 2010). It was discovered and named by the Russian biologist Elie Metchnikoff in 1879 (Zimmermann et al., 1995). According to fungal databases, the genus *Metarhizium* comprises 92 species and is currently classified within kingdom Fungi, subkingdom Dikarya, phylum Ascomycota, subphylum Pezizomycotina, class Sordariomycetes, subclass Hypocreomycetidae, order Hypocreales, and family Clavicipitaceae (Huang et al., 2002). Studies indicates that *Metarhizium* can parasitize more than 200 insect species across 8 orders and 42 families, and can also infect mites and nematodes (Ding et al., 2009; Yang et al., 2015). *M. anisopliae* has been successfully applied in the management of agricultural forestry, and public-health pest, showing measurable control efficacy. It is safe for humans and livestock and is environmentally friendly (Hou Y et al., 2015). This has attracted attention in fields such as plant protection, forestry, and pharmaceutical sciences, indicating broad prospects for further development and application. Studies have shown that has good control efficacy against a variety *Metarhizium anisopliae* of insect pests (Armas et al., 2020; Cai, 2010; Hou Y et al., 2015; Tong et al., 2008; Wan et al., 2023; Wang et al., 2010; Wu and Tong, 2020; Xu and Tong, 2017; Zheng, 2020; Zheng et al., 2024). The target insect species and relevant references are listed in Supplementary Table S1.

The infection of hosts by *Metarhizium* is an extremely complex process. It begins with conidia adhering to the insect's body surface; under certain conditions, the conidia germinate and produce germ tubes, the apical ends of which swell to form a specialized infection structure known as an attachment cell. The attachment cell then grows hyphae or infection spines, which penetrate the insect's cuticle and invade its body (Wang and Wang, 2017). The fungus reproduces by absorbing nutrients from the host while simultaneously destroying its tissues and producing toxins, ultimately leading to the insect's death (Kershaw et al., 1999). Throughout the entire infection process, the formation of the attachment body is crucial (Lv et al., 2008). By generating turgor pressure, the attachment body facilitates the invasion of the host by *Metarhizium* (Cao and Zhu, 2010). The mechanical force generated by intracellular turgor pressure, combined with extracellular enzymes, acts on the insect's cuticle, ultimately achieving infection of the insect (Clarkson and Charnley, 1996). *Metarhizium* produces four proteases: Bacillus protease (Pr1), trypsin (Pr2), cysteine protease (Pr4), and metalloprotease (Pr3) (Meng, 2021). Among these, Pr1 is capable of degrading the insect cuticle (St. Leger et al., 1992). Pr1 plays a role in the initial stage of penetrating the cuticle; it is an extracellular *Bacillus subtilis* protease virulence factor that exerts a major virulence effect and also promotes the germination of *Metarhizium* conidia (Shah et al., 2005). Overexpression of this gene significantly enhances the virulence of *Metarhizium* (St. Leger et al., 1996). Insect cuticular proteins play a role in preventing *Metarhizium* invasion (Wang and You, 1999) and the presence of unoxidized phenols and their metabolites in the interstitial spaces of cuticular macromolecules can also inhibit the germination of *Metarhizium* conidia (Zhou, 1997). Hydrocarbons can enhance insect resistance by strengthening the cuticular barrier (Chen, 2018; Wood et al., 2010). It is known that the hydrocarbon content of the cuticular layer influences fungal pathogenicity, and numerous antimicrobial compounds have been observed on the cuticle. For example, extracts from the cuticle of the European corn borer (*Helicoverpa zea* Boddie) are toxic to *Beauveria bassiana* (Smith and Grula, 1982). The combination of epidermal phenolic compounds and tyrosinase activity leads to melanization, which is typically a response to microbial attacks. Melanin and melanized tissues can inhibit the germination of *Metarhizium* conidia and hyphal growth, provide greater resistance to mechanical penetration and the action of fungal degradative enzymes, and act as a barrier limiting nutrient uptake by pathogens (St. Leger et al., 1998). Protease inhibitors with high inhibitory activity against *Metarhizium* Pr1 protease have been detected in the molting fluid of the tobacco hornworm (*Manduca sexta* Linnaeus) (Samuels and Reynolds, 2002). Most long-chain fatty acids, however, are believed to promote the germination and growth of *Metarhizium* conidia (Wang, 2005). *Metarhizium acridum* Sorokin forms attachment spores only on the body surface of its host, the desert locust (*Schistocerca gregaria* Boris), and does not form them on the body surface of the non-host 17-year cicada (*Magicicada septendecim* Carl). Subsequent studies found that polar extracts from the locust's body surface can induce the production of attachment spores (Wang and St. Leger, 2005). These indicates that the formation of *Metarhizium* acridum attachment spores is dependent on the induction by chemical signals from specific hosts. Research indicates that during host infection of *M. anisopliae*, appressorium formation is significantly and positively correlated with virulence (Mou, 2022). Appressoria not only serve as a prerequisite for colonization of the host cuticle by *M. anisopliae* but also produce proteases, chitinases, and lipases that degrade the insect cuticle, thereby promoting successful infection (Li, 2015; Zhao et al., 2025). Previous studies on appressoria have primarily focused on their physicochemical processes and the chemical effects of appressorium formation on the insect cuticle (Lv et al., 2008; Lai et al., 2020; Sánchez-Pérez et al., 2014; Wang et al., 2023). However, there are comparatively few reports on the effects of insect cuticular compounds on appressorium formation by *M. anisopliae* during infection and on the associated metabolic impacts. Previous studies identified 102 differentially abundant insect cuticular compounds that were specific to the appressorium formation stage of *M. anisopliae*, these compounds were significantly changed in abundance and uniquely enriched during the appressorium developmental period compared with other growth stages of *M. anisopliae* (Xu et al., 2026). In this study, we present a novel paradigm in fungal pathogenesis by moving beyond traditional nutritional perspectives. We systematically screened 102 differential cuticular compounds and selected 10 representatives across six distinct categories-including amino acids, amines, aromatic compounds, organic acids, flavonoids, and glycerophospholipids-to evaluate their specific effects on appressorium formation. The pivotal innovation of our work lies in the strategic selection of insect cuticular metabolite analogues that actively promote appressorium development, combined with LC-MS-based untargeted metabolomics to map the resulting metabolic reprogramming in *M. anisopliae*. This approach uniquely reveals how specific cuticular chemicals act as precise signaling cues to orchestrate fungal development, providing a transformative theoretical basis for elucidating the pathogenic mechanisms of *M. anisopliae* and its chemical interplay with the insect cuticle, and opening new avenues to enhance its infectivity through targeted metabolic manipulation.

## 2 Materials and methods

### 2.1 Primary instruments

Fluorescence microscope (Axio Imager A2, ZEISS, Germany), Critical Point Drying Apparatus (SCD-380A, Shianjia, China), Mass Spectrometer (Orbitrap Exploris 120, Thermo Fisher Scientific, USA), Ultra-high-performance liquid chromatography (UHPLC) system (Vanquish UHPLC, Thermo Fisher Scientific, USA), liquid chromatography column (Waters ACQUITY UPLC BEH Amide, Thermo Fisher Scientific, USA), refrigerated centrifuge (D3024R, Scilogex, USA).

### 2.2 Primary reagents

Insect cuticle compound analogues: Epigallocatechin gallate (99.00%, Glpbio, USA), L-Iiditol (98.00%, Glpbio, USA), Sulfadoxine (99.5%, Glpbio, USA), L-Aspartic acid (98.00%, Glpbio, USA), 4-(Aminomethyl)benzoic acid (98.00%, Glpbio, USA), Fenofibric acid (99.50%, Glpbio, USA), Cholini bitartras (99.00%, Glpbio, USA), 2,6-Dihydroxybenzoic Acid (99.50%, Glpbio, USA), Glycy-L-phenylalanine (98.00%, Glpbio, USA), Miltefosiine (98.00%, Glpbio, USA).

### 2.3 Test strain and conidial suspension preparation

The test strain was *M. anisopliae* MaHA-01 (Wan et al., 2023), first isolated from soil in Huian, Fujian Province in September 2005. Using the immersion method, healthy fourth-instar larvae of the *Opisina arenosella* Walker were immersed up to the head in a suspension of *Metarhizium anisopliae* spores at a concentration of 1 × 108 conidia/mL for 15 s. They were then removed, left to air-dry naturally, and placed in a climate chamber for rearing. Following the larvae had died and their bodies had stiffened, the surface of the carcasses was disinfected by soaking them in 75% ethanol for 1 min to kill surface contaminants, followed by thorough rinsing three times with sterile water to remove residual ethanol. The treated carcasses were placed in petri dishes lined with moistened filter paper for cultivation under humid conditions. Once the surface of the insect carcasses is covered with green conidia, pick out fresh spores and inoculate them onto PDA medium for purification. The above process is repeated three times consecutively, with each repetition constituting one round of recovery. Take the revived strain was inoculated onto potato dextrose agar (PDA) medium (200 g peeled potatoes, 20 g glucose, 20 g agar, brought to 1000 mL with water; pH unadjusted) and cultured in a constant-temperature incubator at (26 ± 1)°C for 7–10 d. Upon sufficient sporulation, conidia were scraped from the medium surface with a sterile loop into sterile flasks containing 10–15 glass beads and sterile water. The flasks was shaken at 150 rpm for 20 min to disperse spore clumps. Conidia were counted using a hemocytometer, and a conidial suspension with a concentration of 1.0 × 108 conidia/mL was prepared for subsequent use.

### 2.4 Determination of conidial germination rate and appressorium formation rate of *M. anisopliae*

In a sterile aqueous solution containing 1 mg/mL yeast extract, cuticular compound analogues showing significant differences were added to prepare 5 concentration-gradient mixtures. Each mixture was used to prepare a *M. anisopliae* conidial suspension at a concentration of 5 × 106 conidia/mL. A *M. anisopliae* suspension with yeast extract concentrations of 1 mg/mL each served as the control. The suspensions were thoroughly mixed by shaking and incubated in shaker at (26 ± 1)°C and 150 rpm. After 12 h, 24 h, 36 h, 48 h, and 72 h of incubation, 100 μL was sampled for microscopic observation, and number of germinated conidia and conidia forming appressoria were determined. For each treatment, five independent biological replicates were prepared. Each replicate was treated with 100 μL of conidial suspension at a concentration of 5 × 106 conidia/mL and examined under a light microscope. For each aliquot, five randomly selected fields of view were photographed, and the numbers of germinated and non-germinated conidia were recorded, with a minimum of 20 conidia counted per field of view. Accordingly, at least 100 conidia were assessed per biological replicate. The germination rate for each replicate was calculated as the mean percentage across the five fields of view. Statistical differences among treatments were evaluated using one-way analysis of variance (ANOVA) followed by Tukey's honestly significant difference (HSD) *post-hoc* test, with the significance level set at α = 0.05. All data are presented as mean ± standard deviation (SD) from five biological replicates. Germination was defined as the emergence of a germ tube exceeding twice the length of the conidium.

### 2.5 Determination of the effects of insect cuticular compound analogues on the metabolism of *M. anisoplae* appressoria

#### 2.5.1 Sample processing

Add *M. anisoplae* conidial powder to a sterile aqueous solution containing 1 mg/mL yeast extract to prepare a conidial suspension with a concentration of 5 × 106 conidia/mL. Add an insect cuticle compound analogue with a pronounced effect on appressorium formation to the conidial suspension. Use a conidial suspension without the cuticular analogue as the control. Mix thoroughly by shaker and incubate at (26 ± 1) °C and 150 rpm. Based on the observations in Section 2.4, culture suspensions were collected before and after appressorium formation. Each sample was filtered through a 0.22 μm membrane filter. A 1000 μL aliquot of the filtrate was transferred to an Eppendorf tube, followed by the addition of 400 μL of 80% (v/v) aqueous methanol. After vortex, the mixture was kept on ice for 5 min. Centrifugation was then performed for 20 min at 15,000 × g and 4 °C. After centrifugation, a portion of the supernatant was diluted with mass spectrometry-grade water to a methanol concentration of 53%. This mixture was centrifuged for 20 min (15,000 × g, 4 °C). The supernatant was collected, diluted with mass spectrometry-grade water to 53% methanol, centrifuged again for 20 min, and the final supernatant was injected into the LC-MS system for analysis (Want et al., 2013).

#### 2.5.2 LC-MS analysis

Chromatographic separation of the samples obtained in Section 2.3.2 was performed using a Vanquish ultra-high-performance liquid chromatography system (Thermo Fisher Scientific) with a Waters ACQUITY UPLC BEH Amide column (2.1 mm × 100 mm, 1.7 μm). LC mobile phase A consisted of water containing 25 mmol/L ammonium acetate and 25 mmol/L ammonium hydroxide. Phase B was acetonitrile. The sample tray was set at 4 °C and the injection volume was 2 μL.

Mass spectrometry conditions: the scan range was m/z 100–1500; electrospray ionization (ESI) source settings were as follows: spray voltage: 3.5 kV; sheath gas flow rate: 35 psi; auxiliary gas flow rate: 10 L/min; capillary temp: 320 °C; S-lens RF level: 60; aux gas heater temp: 350 °C; polarity: positive, negative. The MS/MS secondary scan adopts data-dependent acquisition (DDA).

### 2.6 Data analysis

Data were organized in Excel 2020 (Microsoft, USA) and analyzed using SPSS 25.0 (IBM, USA). Multiple comparisons among treatments with different reagent concentrations and the control were conducted using Duncan's multiple range test. Raw data on *M. anisopliae* secondary metabolites were subjected to format conversion and metabolite annotation, followed by orthogonal partial least squares discriminant analysis (OPLS-DA) (Lv et al., 2008) and Kyoto Encyclopedia of Genes and Genomes (KEGG) pathway enrichment analysis (Howard et al., 1991; Lai et al., 2020; Sánchez-Pérez et al., 2014; Wang et al., 2023; Want et al., 2013).

The calculation formulas are as follows:


$$\begin{array}{l} \text{Germination rate }(\%)=\frac{\text{Number of germinated conidia}}{\text{Total Number of conidia}}\\ \;\times 100 \end{array}$$ (1)

$$\begin{array}{l} \text{Appressorium formation rate }(\%)=\frac{\text{Number of conidia forming appressoria}}{\text{Number of germinated conidia}}\\ \;\times 100 \end{array}$$ (2)

## 3 Result

### 3.1 Conidial germination and appressorium formation in *Metarhizium anisopliae*

**Figure 1:**
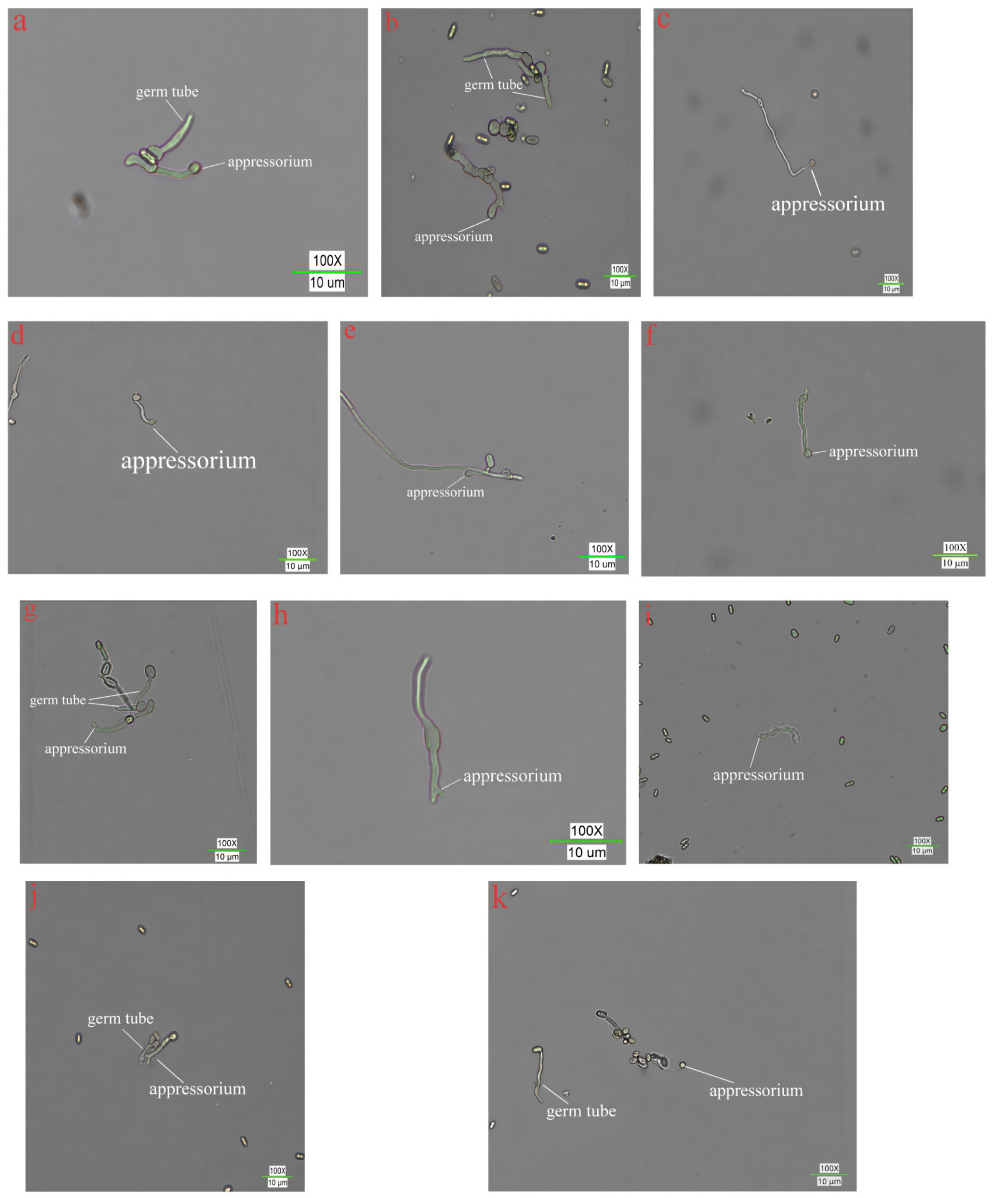
Microscopic images of germ tubes and appressoria formed by conidial germination of Metarhizium anisopliae in the presence of different cuticle compound analogues. **(a)**: Yeast extract; **(b)**: Epigallocatechin gallate; **(c)**: L-Iditol; **(d)**: Sulfadoxine; **(e)**: L-Aspartic acid; **(f)**: 4-(Aminomethyl)benzoic acid; **(g)**: Fenofibric acid; **(h)**: Cholini bitartras; **(i)**: 2,6-Dihydroxybenzoic acid; **(j)**: Glycyl-L-phenylalanine; **(k)**: Miltefosine.

After exogenous addition of analogs of insect cuticle compounds, representative microscopic images of conidial germination and appressorium formation in *M. anisopliae* are presented in Figure 1.

### 3.2 Effects of different insect cuticular compound analogues on conidial germination and appressorium formation in *M. anisopliae*

Following statistical analysis after the adding insect cuticular compound analogues, the results of conidial germination and appressorium formation in *M. anisopliae* are shown in Supplementary Table S2. As shown in Supplementary Table S2, the substances involved were reorganized and analyzed by compound category, we can draw the following conclusions.

#### 3.2.1 Amino acid compounds

L-aspartic acid and glycyl-L-phenylalanine significantly promoted conidial germination at all tested concentrations (*p* ≤ 0.05). Both compounds showed no significant effect on appressorium formation at low concentrations (*p* > 0.05) but significantly enhanced appressorium formation at high concentrations (*p* ≤ 0.05). The maximum germination rate and appressorium formation rate were observed at 1 mg/mL at all time points, which were significantly higher than those of the control (*p* ≤ 0.05).

#### 3.2.2 Amine compounds

L-iditol significantly promoted conidial germination at all concentrations (*p* ≤ 0.05). Low concentrations significantly enhanced appressorium formation within 36 h, whereas high concentrations significantly promoted appressorium formation throughout the entire incubation period (*p* ≤ 0.05). The promoting effect on appressorium formation increased with increasing concentration.

#### 3.2.3 Aromatic compounds

Sulfadoxine and fenofibric acid significantly promoted conidial germination at all concentrations (*p* ≤ 0.05). Sulfadoxine showed no significant effect on appressorium formation only at extremely low concentrations (*p* > 0.05), while fenofibric acid significantly inhibited appressorium formation at extremely low concentrations (*p* ≤ 0.05). At 1 mg/mL, both compounds achieved the highest conidial germination rate and appressorium formation rate, which were significantly higher than those of the control (*p* ≤ 0.05). In contrast, 2,6-dihydroxybenzoic acid significantly inhibited conidial germination and appressorium formation at all concentrations, and the inhibitory effect was strengthened with increasing concentration (*p* ≤ 0.05).

#### 3.2.4 Organic acid compounds

4-(Aminomethyl) benzoic acid significantly promoted conidial germination at all concentrations (*p* ≤ 0.05), and high concentrations also significantly enhanced appressorium formation (*p* ≤ 0.05). Miltefosine significantly promoted conidial germination and appressorium formation only at 0.01 mg/mL, but significantly inhibited both processes at all other concentrations (*p* ≤ 0.05).

#### 3.2.5 Flavonoid and glycerophospholipid compounds

Epigallocatechin gallate (flavonoid) and cholini bitartrate (glycerophospholipid) exhibited dual concentration-dependent effects. Low concentrations significantly promoted conidial germination and appressorium formation, whereas high concentrations significantly inhibited these processes (*p* ≤ 0.05).

Overall, 2,6-dihydroxybenzoic acid and miltefosine showed inhibitory effects on conidial germination and appressorium formation. Epigallocatechin gallate and choline bitartrate promoted growth at low concentrations but inhibited it at high concentrations. In contrast, L-aspartic acid, glycyl-L-phenylalanine, L-iditol, sulfadoxine, fenofibric acid, and 4-(aminomethyl) benzoic acid effectively promoted conidial germination and appressorium formation. Among these, L-aspartic acid displayed the strongest promoting effect.

### 3.3 Effects of t L-aspartic acid on the metabolism of appressoria in *M. anisopliae*

#### 3.3.1 OPLS-DA results

**Figure 2:**
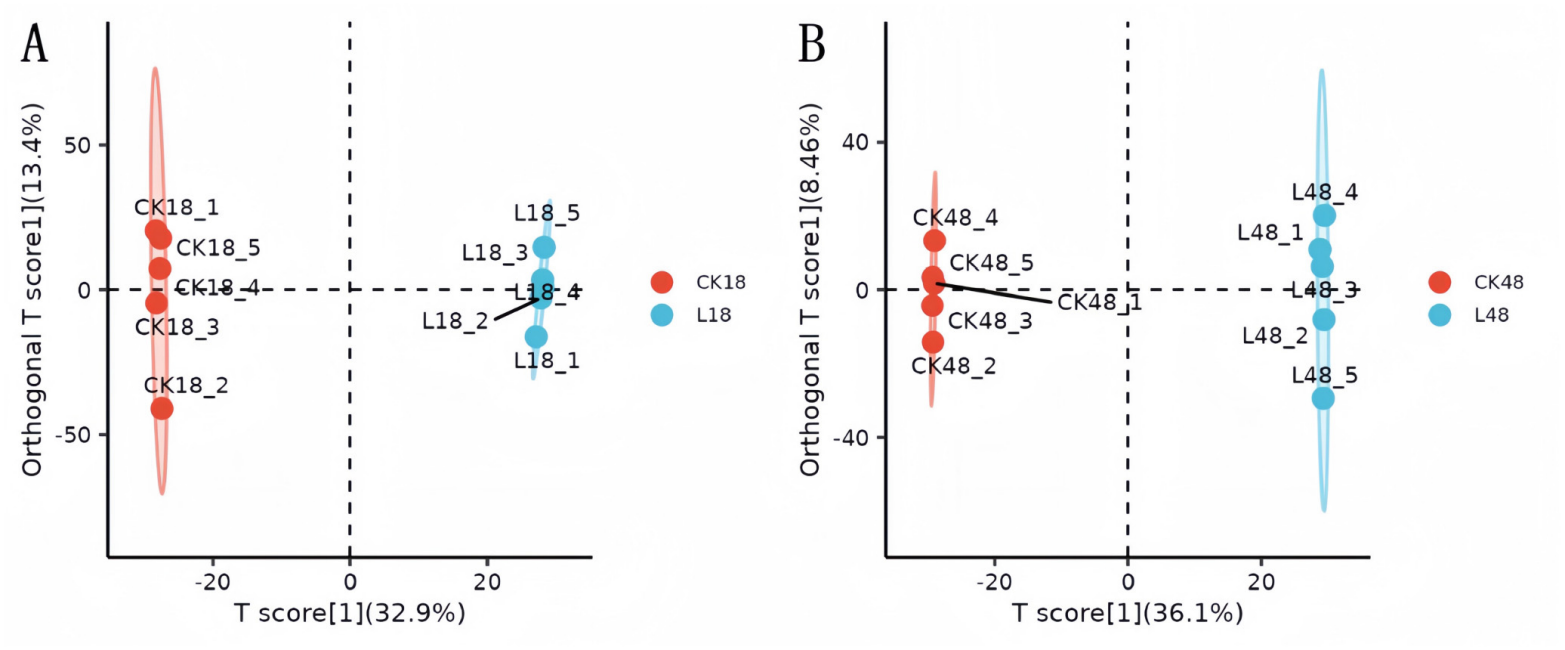
OPLS-DA score plot of secondary metabolites in the L-Asp supplemented treatment group during pre- and post-appressorial formation compared to time-matched controls. **(A)** before appressorium formation, **(B)** after appressorium formation. Among them, A is L-aspartic acid induced after 18 h, B is L-aspartic acid induced after 48 h. The abscissa (x-axis) represents predictive component scores, where separation along this axis reflects inter-group variation. The ordinate (y-axis) displays orthogonal component scores, with dispersion along this axis indicating intra-group variation. Percentages adjacent to axes denote the proportion of dataset variance explained by each component. Model predictive capability is quantified by Q^2^ value, where Q^2^ > 0.5 indicates a statistically valid model.

After the addition of the amino acid-based insect cuticular analogue L-aspartic acid, the metabolic features of *M. anisopliae* before and after appressorium formation were compared with those of the control group at the corresponding time points, and an OPLS-DA score plot was generated; the results are shown in Figure 2. As shown in Figure 2, the metabolic profiles of the treated group both before and after appressorium formation differed significantly from those of the control group. This indicates that, after the addition of L-aspartic acid, the secondary metabolome of *M. anisopliae* was significantly restructured both before and after appressorium formation. The OPLS-DA model demonstrated excellent predictive capability (Q^2^ > 0.5), with its statistical reliability confirmed by cross-validation ANOVA (*p* ≤ 0.05). These findings indicate that L-aspartic acid, an amino acid analog of insect cuticular components, induces a profound reorganization the secondary metabolite profile of *M. anisopliae*. Concurrently, the OPLS-DA model provides a basis for identifying development-associated differentially enriched metabolites in *M. anisopliae*, thereby enabling further metabolite screening based on this model.

#### 3.3.2 Volcano analysis

**Figure 3:**
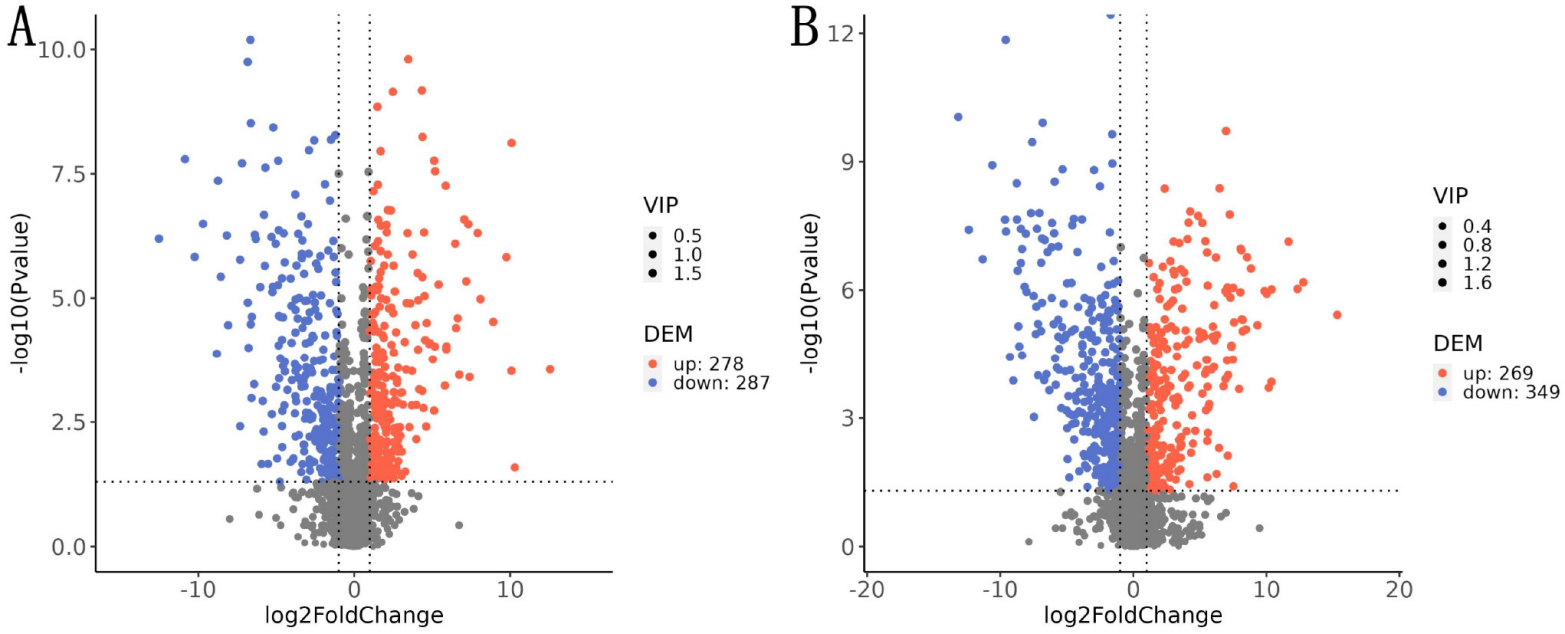
Volcano plot of differential secondary metabolites in the L-Asp supplemented treatment group during pre- and post-appressorial formation compared to time-matched controls. **(A)** before appressorium formation, **(B)** after appressorium formation. Among them, A is L-aspartic acid induced after 18 h, B is L-aspartic acid induced after 48 h. In the volcano plot, each point represents a metabolite. Significantly upregulated metabolites are denoted by red symbols, while significantly downregulated metabolites are indicated by blue symbols. Symbol size scales with the variable importance in projection (VIP) score. The abscissa (x-axis) displays log2-transformed fold-change values, where larger absolute values indicate greater differential abundance between experimental groups. The ordinate (y-axis) shows-log10 (*p*-value), with higher values reflecting greater statistical significance of differential expression.

Using a volcano plot, the overall distribution of differential secondary metabolites between the L-aspartic acid–treated and control groups of *M. anisopliae* before and after appressorium formation is shown in Figure 3. As shown in Figure 3, prior to appressorium formation, 287 of the 565 differential secondary metabolites between the L-aspartic acid-treated group and the control group were significantly upregulated, while 278 were significantly downregulated. After appressorium formation, there were 618 differential secondary metabolites between the L-aspartic acid-treated and control groups, including 268 significantly upregulated and 349 significantly downregulated metabolites. Taken together, following the addition of the amino acid compound L-aspartic acid, significant differential metabolites were observed both before and after conidiophore formation. This indicates that L-aspartic acid broadly perturbs the secondary metabolism of *M. anisopliae*.

#### 3.3.3 Differential metabolites of M. anisopliae before and after appressorium formation following L-aspartic acid treatment

**Figure 4:**
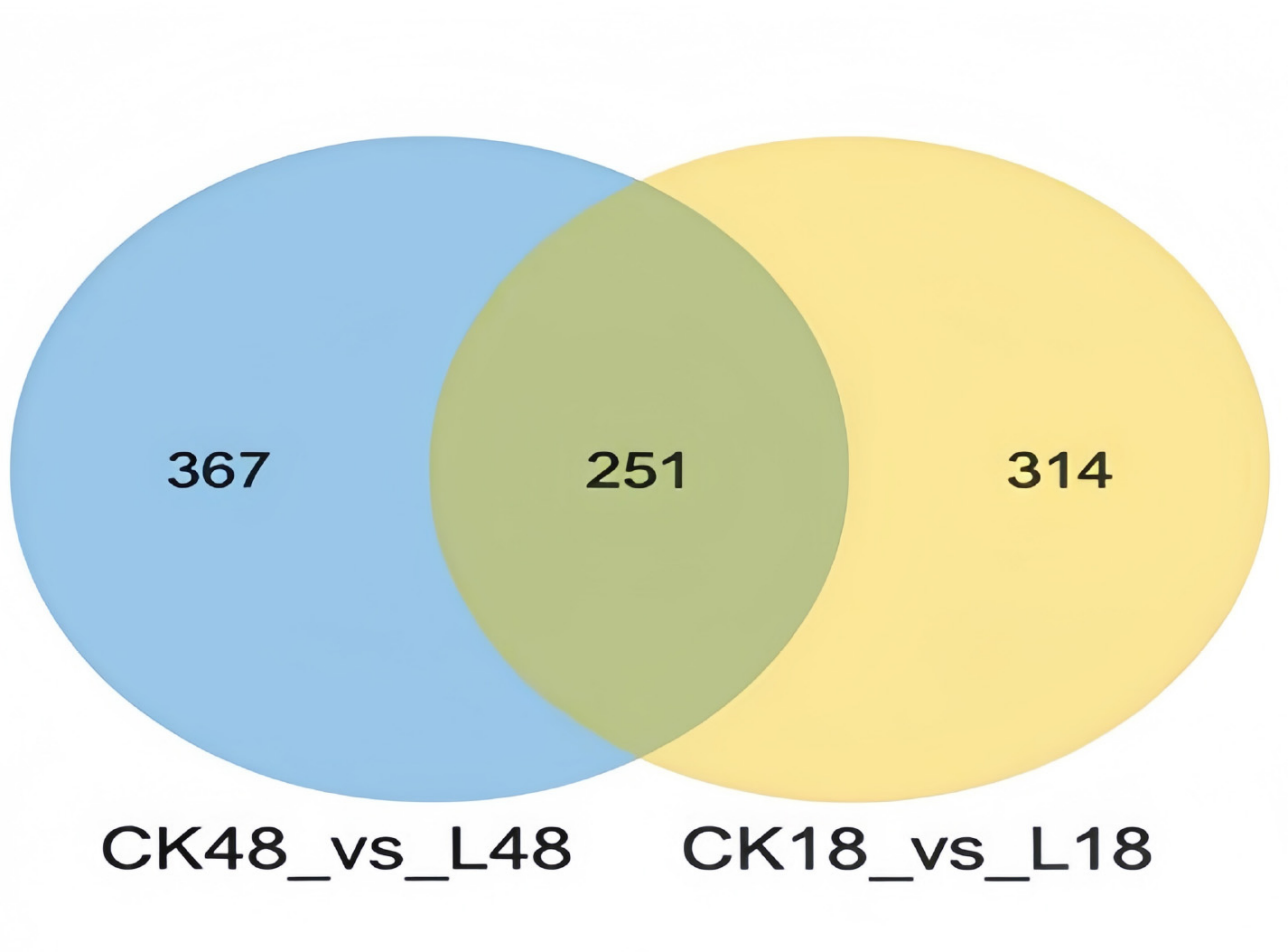
Venn diagram of differential metabolites in the L-Asp supplemented treatment group during pre- and post-appressorial formation compared to time-matched controls.

A Venn diagram was constructed to compare the differential secondary metabolites between L-aspartic acid-treated and control groups of *M. anisopliae* before and after appressorium formation, the results are shown in Figure 4. As depicted in Figure 4, following L-aspartic acid addition, 314 unique differential metabolites were identified before appressorium formation, whereas 367 unique differential metabolites were detected after appressorium formation, and 251 differential metabolites were common to both stages.

**Figure 5:**
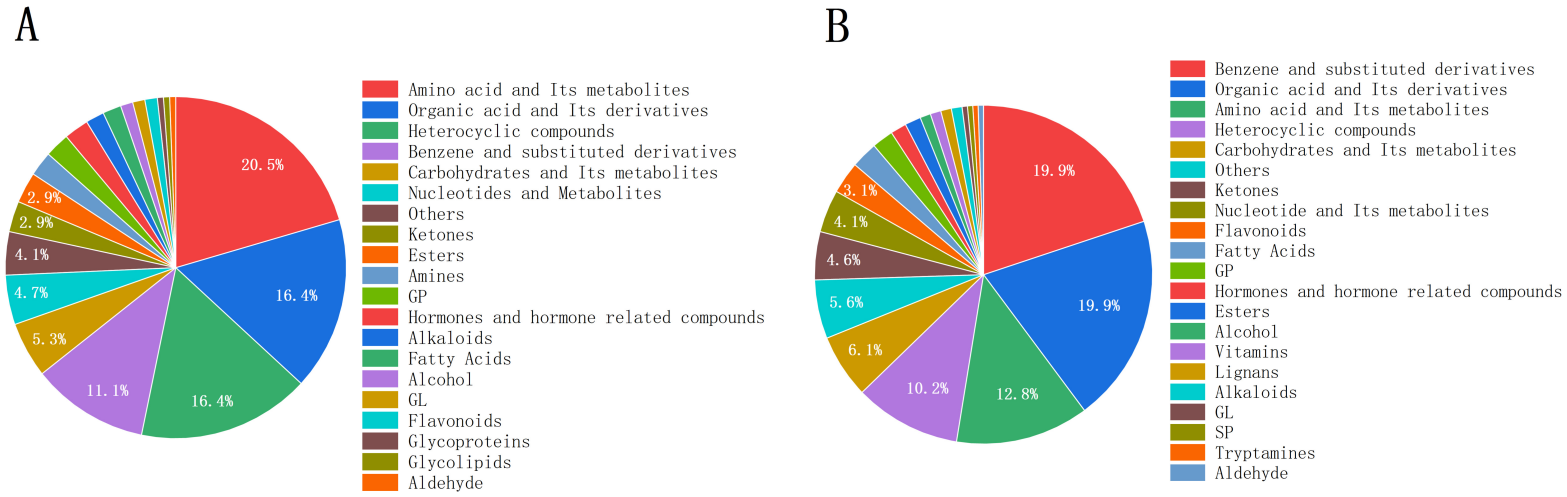
Categories of differential metabolites specific to the L-Asp supplemented treatment group during post-appressorial formation. **(A)** upregulated differential metabolites; **(B)** downregulated differential metabolites.

The 367 unique differential metabolites specific to the post-appressorium-formation stage are shown in Figure 5. The upregulated differential metabolites were mainly amino acids and their metabolites, organic acids and their derivatives, and heterocyclic compounds. Among these, amino acids and their metabolites showed the most pronounced upregulation, accounting for 20.5% of the total, and included 35 compounds, such as lysine-threonine, isoleucine-phenylalanine, arginine-cysteine, isoleucine-leucine, and 4-hydroxy-L-tryptophan. Organic acids and their derivatives ranked second (16.4%), comprising 28 compounds, mainly 6-aminopenicillanic acid, oxytetracycline, aminomalonic acid, DL-pantothenic acid, and tartaric acid. Downregulated differential metabolites primarily comprised benzenoid compounds and their substituted derivatives, organic acids and their derivatives, and amino acids and their metabolites. Benzenoid compounds and their substituted derivatives accounted for 19.9%, including trifluralin, letrozole, toluenesulfonylurea, 1-hydroxyanthraquinone, and 2,3-dimethoxyphenol among 39 compounds. Organic acids and their derivatives constituted 19.9%, including (S)-ureidoacetic acid, 3-hydroxymethylglutaric acid, 5-acetamidopentanoic acid, glucose-1-phosphate, and L-dihydrolactic acid, among 39 compounds. Amino acids and their metabolites followed, accounting for 12.8%, including 25 compounds, such as L-malic acid, aspartylcysteine, N, N-dimethyl-L-arginine, serine-tryptophan, and histidylglutamine.

#### 3.3.4 KEGG pathway analysis

**Figure 6:**
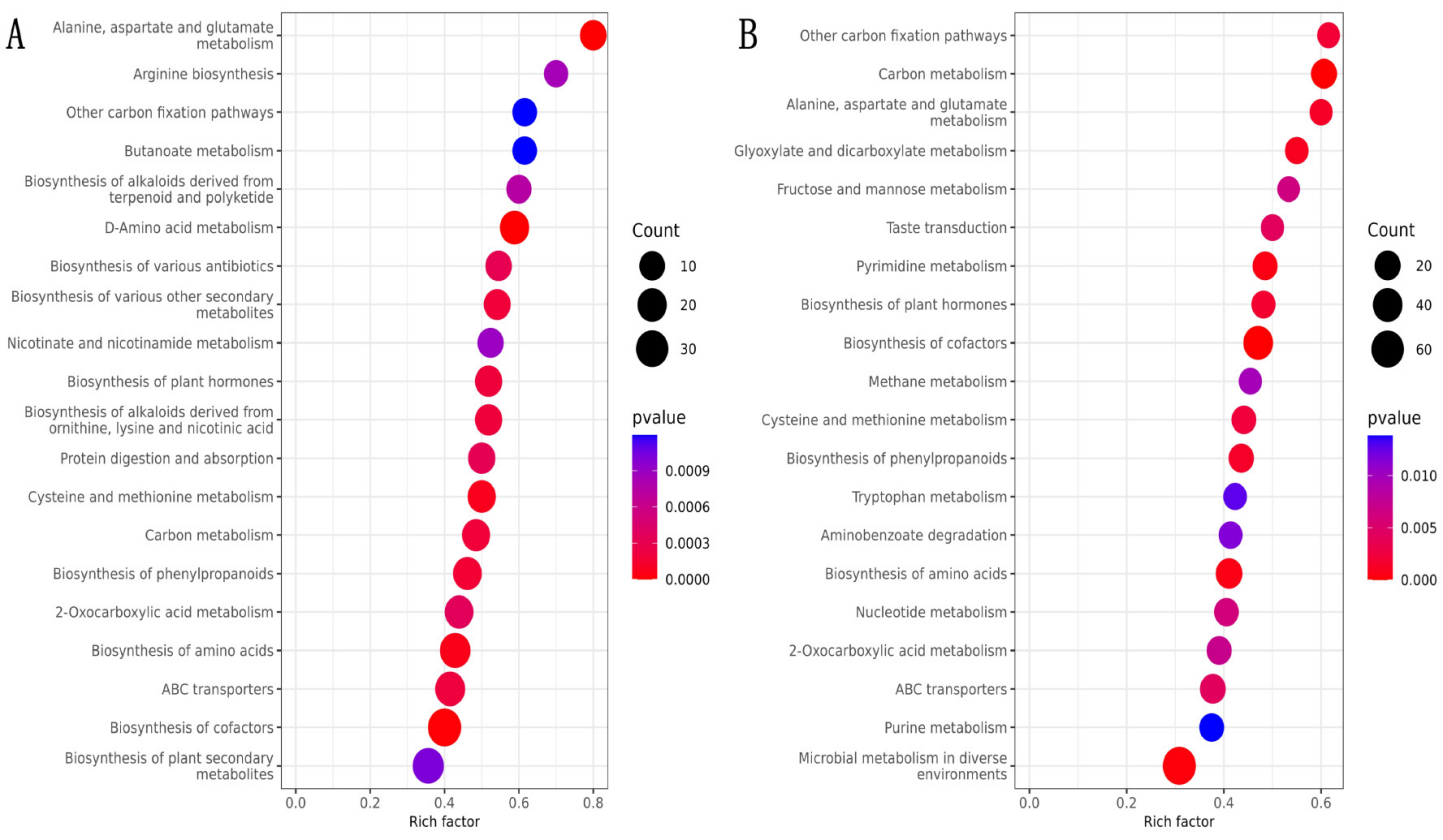
Enrichment analysis of differential secondary metabolites in the L-Asp upplemented treatment group during pre- and post-appressorial formation compared to time-matched controls. **(A)** before appressorium formation, **(B)** after appressorium formation. Among them, A is L-aspartic acid induced after 18 h, B is L-aspartic acid induced after 48 h.

As shown in Figure 6, in the treatment group supplemented with the amino acid compound L-aspartic acid, the differential secondary metabolites of *M. anisopliae* prior to appressorium formation were enriched in 218 metabolic pathways compared with the control group; 89 metabolic pathways were unique to the pre-appressorium stage when compared with the post-appressorium stage, mainly involving D-amino acid metabolism, cofactor biosynthesis, amino acid biosynthesis, cysteine and methionine metabolism, and phenylpropanoid biosynthesis. These pathways are mainly involved in carbon and nitrogen metabolism, energy supply, reducing power generation and stress adaptation, serving as the core metabolic basis by which L-aspartic acid promotes spore germination and early appressorium development. After appressorium formation, the differential metabolites between the treatment and control groups enriched 101 metabolic pathways. Compared to the pre-conidiophore stage, 15 metabolic pathways were unique to the post-appressorium stage when compared with the pre-appressorium stage, mainly involving tryptophan metabolism, the two-component system, β-alanine metabolism, and folate metabolism. These pathways regulate mycelial growth, signal transduction, morphological transition and toxin synthesis, constituting the key metabolic mechanism by which L-aspartic acid enhances the infectivity of *M. anisopliae*. Additionally, both groups shared enrichment across 5 metabolic pathways, including biosynthesis of amino acid and phenylpropanoid biosynthesis, cysteine and methionine metabolism, and other carbon fixation pathways, which not only sustain basal biomass but also confer rapid reprogramming and ecological adaptability under fluctuations in nutrient availability, light, and abiotic stress. The results indicate that the L-aspartic acid-treated group exhibited significantly higher growth efficiency than the control group, and differential pathway analysis revealed that it accelerates signal output via carbon cycling, created an antimicrobial microenvironment through the phenylpropanoid pathway, and immediately activates appressorium-associated genes through the plant hormone biosynthesis; simultaneously, supported by a carbon-concentrating mechanism within the non-canonical carbon-fixation module, it continuously supplies ATP and reducing equivalents to invasive structures. Thereby rapidly establishing infection sites before appressorium formation and after formation, maintaining this structure through metabolic reinforcement and quorum sensing. Ultimately enhancing fungal infectivity and expansion throughout this process.

## 4 Discussion

This study evaluated the effects of 10 analogues of *Opisina arenosella* larvae cuticular compounds on the germination of *M. anisopliae* conidia and the formation of appressoria. The results showed that these 10 analogues significantly influenced the germination of *M. anisopliae* conidia and the rate of appressoria formation in scarab beetles. Among these, the insect cuticle amino acid compound L-aspartic acid at a concentration of 1 mg/mL significantly promoted spore germination and appressorium formation in *M. anisopliae* (*p* ≤ 0.05). Specifically, the appressorium serves as the primary infection structure for entomopathogenic fungi. Its apical region differentiates into a conical penetration peg, which generates transient mechanical thrust through intracellular turgor pressure reaching 6–8 MPa. This force mechanically pierces the host cuticle and promotes epidermal penetration via the secretion of extracellular enzymes such as proteases, chitinases, and lipases, and formation rate of appressoria shows a significant positive correlation with virulence (Howard et al., 1991). L-aspartic acid provides energy and building blocks for fungal metabolic activities (Yang and Min, 2016). Studies have shown that fly larvae powder, cicada exuviae and small amounts of cockroach cuticle contains aspartic acid (Aniebo et al., 2008; Fan, 1985; Jensen et al., 1997). For *M. anisopliae*, adding fly larvae powder, cicada exuviae, or small amounts of cockroach cuticle powder to a medium containing 0.0125% YEM significantly promoted appressorium formation, indicating the presence of active components in the host body wall that induce entomopathogenic fungal differentiation (Li, 2003). The findings of this study are consistent with these observations. This finding provides a theoretical basis for enhancing the virulence of fungal insecticides.

As appressoria serve as the representative infective structure of *Metarhizium anisopliae*, the profiling of differential metabolites at this developmental stage can reveal the regulatory mechanism of fungal virulence. This study employed LC-MS for metabolomic analysis and found that, following the addition of the amino acid L-aspartic acid, 565 differentially expressed metabolites in the pre-appressorium formation stage of *Metarhizium anisopliae* compared to the control group were primarily enriched in pathways such as D-amino acid metabolism, cofactor biosynthesis, amino acid biosynthesis, cysteine and methionine metabolism, and phenylalanine biosynthesis. These pathways are primarily involved in the growth and development of *M. anisopliae*, host infection, environmental adaptation, and virulence formation. Among these, D-amino acid metabolism helps maintain cell wall stability and enhances stress adaptation (Yang et al., 2023); cofactor biosynthesis supports energy metabolism and various virulence-related enzymes (Sheng et al., 2022); amino acid biosynthesis serves as a crucial foundation for hyphal growth, spore formation, and secondary metabolite synthesis (Yang et al., 2024); cysteine and methionine metabolism enhance antioxidant capacity and gene regulation through glutathione synthesis and methylation (Pang et al., 2023); while phenylalanine biosynthesis is closely associated with melanin formation, stress resistance, and host interactions (Qin and Xia, 2024). The significant enrichment of these pathways typically indicates that *Metarhizium anisopliae* is in a state of active metabolism, enhanced virulence, and adaptation to environmental stress. After supplementation with the amino acid L-aspartic acid, a total of 618 differential metabolites were identified between the appressorium-forming group of *M. anisopliae* and the control group. These metabolites were mainly enriched in the tryptophan metabolism, two-component system, folate metabolism and β-alanine metabolism pathways. Among them, the tryptophan metabolism pathway can provide nutrients for fungi or act as signaling molecules to regulate their growth. Tryptophan metabolic pathways can provide nutrients to fungi or influence their growth by acting as signaling molecules. For example, indole compounds produced through tryptophan metabolism can regulate cellular signaling pathways, promoting cell division and growth (Wang et al., 2021). Similarly, kynurenine can scavenge reactive oxygen species and maintain cellular redox balance; under cold stress, related metabolites can prevent lipid peroxidation and protect cell membrane integrity (Chen et al., 2023). Furthermore, some fungi can synthesize toxic secondary metabolites through tryptophan metabolism. For example, the *Diaporthe* sp. AC1, when induced by 1-methyl-L-tryptophan, produces antimicrobial and cytotoxic secondary metabolites that play a crucial role in the fungal pathogenic process (Zhang, 2023). Two-component system metabolic pathways play a crucial role in fungal morphogenesis and the expression of virulence factors. For instance, the Hog1 MAPK pathway in *Candida albicans* Berkhout, as part of a two-component system, responds to oxidative stress, regulates respiratory metabolism, and influences virulence (Hou B B et al., 2015). Similarly, the GacS/GacA two-component system in *Pseudomonas* species regulates secondary metabolism and signal transduction, influencing their pathogenicity toward plants (You et al., 2024). Furthermore, two-component systems also regulate fungal morphological transitions (such as from the yeast to the hyphal state), which are critical for colonization and pathogenicity within the host (Hou B B et al., 2015). The folate metabolism pathway is critical for fungal pathogenicity. During fungal infection, the expression of genes related to folate metabolism is upregulated, aiding in rapid fungal proliferation and the maintenance of pathogenicity (Yan et al., 2023). Furthermore, the folate metabolism pathway is associated with the synthesis of fungal secondary metabolites, which play important roles in virulence and adaptability. For example, some filamentous fungi enhance their survival within the host by regulating the folate metabolism pathway (Pan and Liu, 2018). In the interaction between fungi and hosts, the upregulation of the folate metabolism pathway helps fungi adapt to the host immune environment and regulates pathogenicity through mechanisms such as epigenetic modifications (Yan et al., 2023). The β-alanine metabolism pathway is closely associated with the synthesis of fungal secondary metabolites. For instance, in *Penicillium chrysogenum* Christiaan, 1,3-diaminopropane (1,3-DAP) can induce the synthesis of β-alanine and pantothenic acid, thereby activating the biosynthesis of secondary metabolites such as penicillin (Martín and Liras, 2024). This metabolic regulatory mechanism plays a crucial role in the production of antibiotics and other bioactive compounds by fungi. Furthermore, β-alanine metabolism helps fungi resist oxidative stress and enhances their survival in adverse environments (Nie et al., 2023). β-alanine metabolism also indirectly influences the expression of fungal virulence factors and their ability to survive and reproduce within the host by regulating secondary metabolite synthesis, intracellular pH, and redox balance (Martín and Liras, 2024). These pathways drive the coordination of “fungal growth regulation-morphological transition-toxin synthesis” (Bahn, 2008; Li et al., 1998; Pollmann et al., 2009; Wang et al., 2014) and ultimately enhancing the overall infection efficiency.

Studies have shown that the secondary metabolism of *M. anisopliae* is not only influenced by nutritional conditions but is also key to host-specific adaptation. During the early stages of infection upon contact with the arthropod cuticle, the fungus rapidly adjusts its metabolic strategy by upregulating specific biosynthetic gene clusters (BGCs), including those encoding pseurotin and helvolic acid (Sbaraini et al., 2016). This suggests that *M. anisopliae* possesses a sophisticated “chemical reconnaissance” system capable of recognizing cuticle components (such as L-Aspartic acid) and initiating the infection process. Following apressorium formation, 367 unique differential metabolites were identified, including organic acids and their derivatives, benzenoid compounds and their substituted derivatives, as well as amino acids and their metabolites. Organic acids and their derivatives play crucial roles during *M. anisopliae* growth by regulating pH, and appressorium formation requires substantial energy; glycerol and fatty acids generated by lipid droplet degradation provide an osmotic turgor pressure (Li et al., 2022). Concurrently, shifts in organic acid concentrations can serve as indicators of intracellular energy status, thereby promoting appressorium development (Wang and St. Leger, 2007), and intermediates of organic acid metabolism participate in regulating *M. anisopliae* sporulation capacity (Yang et al., 2024). Benzenoid compounds and their substituted derivatives may act as chemical signals that trigger *M. anisopliae* appressorium differentiation. After spore-surface hydrophobins sense cuticle-associated benzenoid compounds, the cAMP-PKA signaling pathway is activated, inducing appressorium-specific gene expression and morphogenesis; this mechanism is well supported in *Beauveria bassiana* and is conserved in *M. anisopliae* (Wang and St. Leger, 2007). In additionally, benzenoid compounds may also exhibit antimicrobial activity, aiding *M. anisopliae* compete more effectively for resources in natural environments (Fan et al., 2017; Pan and Liu, 2018; Xu et al., 2008). Amino acids and their metabolites serve as nitrogen sources for protein synthesis, promoting mycelial growth and spore formation, and are essential nutrients for *M. anisopliae* growth (Yang et al., 2024). These changes indicate that L-aspartic acid significantly impacts the secondary metabolism of *M. anisopliae* during the appressorium formation stage, and the functions of the following three differential metabolites warrant further investigation: (1) Nicotinamide mononucleotide (NMN) is an intermediate in NAD synthesis, and NAD provides energy for *M. anisopliae* (Yang and Sauve, 2016; Sant'Anna et al., 2020). NAD and its metabolites regulate signaling pathways associated with growth, development, and stress responses in cellular signaling. In additionally, NAD modulates cellular metabolism, gene expression, and stress tolerance by serving as a substrate for deacylases (Amjad et al., 2021; Xie et al., 2020). Thus, NMN may modulate *M. anisopliae* signaling and function via NAD. Furthermore, in certain medicinal fungi, nucleotides promote mycelial growth and fruiting body development (Zhang et al., 1995). It is hypothesized that NMN supplies energy and signals to *M. anisopliae*, stimulating mycelial elongation and branching to expand colonies. Concurrently, it may stimulate spore production, enhance yield, and facilitate its development and dissemination. (2) Isoleucine and phenylalanine participate in the synthesis of structural proteins, glycoproteins, and enzymatic proteins, serving as key substrates for protein synthesis in numerous microorganisms (Han et al., 2025). They contribute to the synthesis of conidial wall proteins in *M. anisopliae*, thereby influencing conidial wall structure and stability; during germination, they rapidly supply energy and nitrogen, activating enzymatic activity and protein synthesis to drive germ tube elongation (Fang et al., 2009). Studies have reported that isoleucine deficiency inhibits sporulation in *Bacillus subtilis* Christian (Errington, 2003), therefore, this amino acid is inferred to promotes spore germination and hyphal elongation in *M. anisopliae*. (3) 1-Hydroxyanthraquinone can disrupt the insect endocrine system, affecting the synthesis, secretion, or signal transduction of ecdysone. For example, anthraquinone compounds extracted from *Morinda citrifolia* L. significantly decrease ecdysone levels in mosquitoes, leading to developmental arrest in larvae (Kamiyama and Niwa, 2022; Hun et al., 2022). Alizarin (an anthraquinone compound) can directly bind to the ecdysone receptor (EcR), blocking the 20-hydroxyecdysone (20E) signaling pathway and inhibiting pupation and eclosion in *Drosophila melanogaster* Johann (Pitoniak and Bohmann, 2015). Simultaneously, 1-hydroxyanthraquinone disrupts insect cell membrane integrity, interferes with cellular respiration and metabolism, acts on the insect nervous system, and impairs nerve impulse transmission, leading to paralysis, convulsions, and even death in insects (Zhu et al., 2022). Furthermore, microbial communities exert important influences on insect growth, development, and immune function, and 1-hydroxyanthraquinone shows inhibitory activity against certain bacteria and fungi (Rolta et al., 2020). It is hypothesized that 1-hydroxyanthraquinone synthesized by *M. anisopliae* indirectly affects insect health by suppressing the growth and proliferation of symbiotic microorganisms, thereby rendering insects more susceptible to environmental stressors and pathogen invasion. The amino acid compound L-aspartic acid in the insect cuticle induces secondary metabolism in *M. anisopliae*, significantly enhancing its infectivity; the underlying mechanism can be summarized in three points: (1) L-aspartic acid promotes the biosynthesis and secretion of toxins or acidic substances by *M. anisopliae*, disrupting host ecdysone receptor signaling and intestinal pH homeostasis. This causes insect developmental arrest and physiological disturbance, facilitating hemolymph dissemination of *M. anisopliae* and creating favorable conditions for fungal invasion; (2) L-aspartic acid markedly increases the appressorium formation rate of *M. anisopliae* by promoting activation of tryptophan metabolism and a two-component system signaling cascade. It concurrently induces oxidative-stress pathways, providing immediate energy and reducing power for turgor generation in the penetration peg, thereby enabling mechanical breach of the insect cuticle and facilitating *M. anisopliae* invasion. (3) L-aspartic acid promotes the production of antimicrobial substances by *M. anisopliae*, suppressing colonization of insect symbiotic microbial communities and weakening both cellular and humoral immunity. This collectively reduces host defensive efficacy against *M. anisopliae*, thereby enhancing fungal infectivity and ecological competitiveness.

## 5 Conclusion

Research indicates that: (1) At certain concentrations, the insect cuticle amino acid compounds L-aspartic acid and glycyl-L-phenylalanine promote spore germination and appressorium formation in *M. anisopliae*, thereby facilitating its infection of the host; (2)The amino acid compound L-aspartic acid significantly influences metabolism in *M.anisopliae*. Based on this finding, we propose a temporal metabolic switch model, revealing that L-Asp acts as a master regulator that orchestrates the transition from biosynthesis-dominated growth to infection-dominated penetration via specific metabolic cascades.

### 5.1 Phase 1: biosynthesis-dominated phase (before appressorium formation)

During the early stages of *Metarhizium* antheridial germination and pre-adherency, L-aspartic acid triggers a metabolic shift that takes precedence over cell proliferation and stress recovery. Metabolic pathways significantly enriched during this stage include D-amino acid metabolism, cofactor biosynthesis, cysteine and methionine metabolism, and phenylalanine biosynthesis. This series of changes lays the “metabolic foundation” for subsequent morphological differentiation. Specifically, the upregulation of biosynthetic pathways provides the essential precursors (such as amino acids and nucleotides) and energy (ATP) required for rapid cell division. Concurrently, the activation of antioxidant defense compounds (such as phenylpropanoids) enables meristems to effectively withstand oxidative bursts and physical barriers on the host surface, thereby successfully initiating subsequent morphological transformations.

### 5.2 Phase 2: infection-dominated phase (after appressorium formation)

Upon completion of adherent cell differentiation, metabolic flux is redirected toward host invasion and parasitism. Its primary characteristics include a shift in metabolic pathways toward tryptophan metabolism, the two-component system, β-alanine metabolism, and folate metabolism. This stage implements a strategy of “osmotic penetration and host suppression.” Regarding mechanical penetration, activation of the two-component system likely regulates the signaling cascade required to generate massive osmotic pressure within the adhezoite. This pressure drives the formation of penetration pegs, thereby achieving a physical breach of the host epidermal barrier. Concurrently, the surge in secondary metabolites (such as 1-hydroxyanthraquinone) and virulence factors serves to chemically neutralize host defenses. These metabolites may disrupt the host's molting and developmental processes by interfering with the ecdysone receptor (EcR) signaling pathway, while also suppressing competing microorganisms, thereby ensuring the fungus's colonization advantage

This model delineates how L-Asp acts not merely as a nutrient source, but as a master regulator orchestrating the fungal metabolic landscape. The exact mechanisms by which L-aspartic acid influences spore germination, appressorium development, and the resulting enhancement in pathogenicity remain to be fully determined. Nevertheless, this work offers a valuable perspective on fungal interactions with the insect cuticle. It provides a theoretical basis that may contribute to a deeper understanding of *M. anisopliae* pathogenesis and the refinement of its application in biological control.

## Data Availability

The original contributions presented in the study are included in the article/Supplementary material, further inquiries can be directed to the corresponding author/s.
