# Supplementary material for: Effect of insect cuticular compounds on appressorium formation and metabolic activity in Metarhizium anisopliae
Source: Front Microbiol. 2026 Jun 18;17:1842196. doi: 10.3389/fmicb.2026.1842196 (PMC13326655; doi:10.3389/fmicb.2026.1842196)
Supplement: Supplementary file 1 [file Table_1.pdf]

Table S1: Insect pests controlled by *Metarhizium anisopliae*

No.	Insect pest	Reference
1	*Hylobitelus xiaoi* Zhang	Tong et al., 2008
2	*Lymantria xylina* Swinhoe	Cai, 2010
3	*Spathius anoplophorae* Yang	Wang et al., 2010
4	*Locusta migratoria manilensis* Meyen	Hou Y et al., 2015
5	*Brontispa longissima* Gestro	Xu and Tong, 2017
6	*Hyphantria cunea* Drury	Armas et al., 2020
7	*Solenopsis invicta* Buren	Wu and Tong, 2020
8	*Milionia basalis* Walker	Zheng, 2020
9	*Opisina arenosella* Walker	Wan et al., 2023
10	*Monochamus alternatus* Hope	Zheng et al., 2024

Table S2: Spore germination rate and appressorium formation rate of *Metarhizium anisopliae* in different concentrations of epidermal compound analogue

Subtance	Concentration (mg/mL)	Germination rate at 12 h (%)	Germination rate at 24 h (%)	Appressorium differentiation rate at 24 h (%)	Germination rate at 36 h (%)	Appressorium differentiation rate at 36 h (%)	Germination rate at 48 h (%)	Appressorium differentiation rate at 48 h (%)	Germination rate at 72 h (%)	Appressorium differentiation rate at 72 h (%)
Epiigallocatechin gallate	0.01	23.40±1.67a	46.80±2.28b	3.20±0.84c	59.40±2.61ab	23.80±1.64a	74.80±2.28a	43.60±2.70a	86.40±1.67a	49.60±2.41a
	0.05	20.00±2.24b	52.00±2.45ab	5.00±0.71b	56.20±1.30b	19.20±0.84b	73.80±2.18a	38.40±3.21b	83.40±2.97b	47.20±2.05a
	0.1	12.00±2.24c	32.80±1.79c	12.00±1.58a	44.8±2.49c	12.40±1.67c	65.20±3.03b	28.00±3.94c	74.60±3.36c	33.40±2.70c
	0.5	1.80±0.84d	3.40±0.55d	0d	5.00±0.71d	0d	15.40±1.67c	0d	18.40±1.34d	0d
	1	0d	0.00±0.00d	0d	0e	0d	0d	0d	0e	0d
	CK	24.80±3.42a	55.20±10.08a	5.60±1.14b	62.50±3.85a	18.6 ±1.95b	65.8±4.97b	28.40±2.07c	76.20±2.18c	36.4±2.30b
L-Iditol	0.01	18.00±1.22c	52.40±1.52c	4.40±0.55b	71.80±2.95c	13.60±1.14d	77.00±2.74b	25.60±1.82c	83.00±2.74b	31.80±1.30c
	0.05	20.60±2.41c	61.60±1.52b	5.80±0.45bc	74.20±2.28bc	15.60±1.82d	75.80±2.90b	27.60±1.52cd	85.00±2.12b	32.80±1.79c
	0.1	27.40±1.52b	63.00±2.12b	7.00±0.71b	76.20±3.11b	24.80±1.10b	79.20±2.77b	29.20±2.17c	84.40±2.61b	32.40±0.89c
	0.5	31.00±1.22a	79.20±3.63a	9.80±1.48a	85.20±1.79a	29.40±2.70a	87.40±4.28a	36.80±1.64b	89.40±2.88a	38.80±1.79b
	1	26.00±1.22b	77.40±2.07a	10.80±1.64a	86.00±2.35a	25.20±1.64b	91.40±3.05a	41.40±1.52a	90.60±2.61a	48.00±2.00a
	CK	24.80±3.42b	55.20±10.08c	5.60±1.14bc	62.00±5.3d	18.60±1.95c	65.80±4.97c	28.40±2.07c	76.20±2.17c	36.40±2.30c

Sulfadoxine	0.01	18.00±1.00c	51.80±2.59ab	8.20±1.30a	65.60±1.67c	17.60±1.95d	74.00±3.74b	23.60±3.43d	80.00±4.00b	34.80±1.79d
	0.05	18.60±1.52cd	62.20±1.640a	7.80±1.48a	73.29±1.64b	18.20±0.84cd	77.60±1.67b	26.40±1.67cd	79.60±2.41bc	40.00±2.00bc
	0.1	21.60±2.51bc	60.00±2.00c	7.40±0.89ab	73.49±2.30b	20.60±2.30c	82.40±2.40a	26.80±1.48c	82.40±1.34ab	38.80±4.87bc
	0.5	22.80±2.05b	52.40±1.95c	7.00±1.22a	78.69±1.14a	25.20±1.10b	84.60±2.88a	32.40±1.67b	84.00±2.92a	41.80±2.28b
	1	32.80±3.56a	64.00±5.15a	7.80±0.84a	81.69±1.34a	33.00±2.00a	85.20±2.68a	39.80±2.77a	86.00±2.45a	46.20±3.0a
	CK	24.80±3.42b	55.20±10.08bc	5.60±1.14b	62.50±3.85d	18.60±1.95cd	65.80±4.97c	28.40±2.07c	76.20±2.17c	36.40±2.30d
L-Aspartic acid	0.01	27.40±2.61c	52.60±1.67d	4.40±1.52d	68.80±2.68c	14.40±1.34e	81.80±2.17c	26.60±1.81d	84.20±1.92d	36.60±1.82d
	0.05	34.20±2.28b	56.20±3.70d	5.60±2.07d	82.20±1.79b	18.40±1.140d	83.80±2.39c	33.20±2.28c	84.60±1.67d	40.60±3.21c
	0.1	36.80±2.86b	64.00±2.459c	8.20±1.30c	83.80±2.28b	21.60±2.30c	87.80±2.17b	33.20±3.49c	87.20±1.92c	42.40±2.07c
	0.5	46.00±2.55a	74.80±1.10b	15.00±1.41b	85.00±2.74 b	27.80±1.10b	92.40±0.89a	37.40±1.67b	92.60±2.07b	47.60±2.61b
	1	49.20±2.59a	86.40±2.41a	18.60±1.67a	95.20±1.48a	39.00±1.22a	94.00±1.22a	65.20±1.79a	95.40±1.95a	66.80±1.10a
	CK	24.80±3.42c	55.20±10.08d	5.60±1.14d	62.00±5.39d	18.60±1.95d	65.80±4.97d	28.40±2.07d	76.20±2.17e	36.40±2.30d
4-(Aminomethyl)benzoic acid	0.01	27.60±1.52b	60.00±2.24c	7.40±0.55d	81.80±2.17b	17.40±1.52d	85.40±1.52c	27.60±1.34e	85.20±2.28b	34.00±1.7d
	0.05	28.80±1.64b	73.00±1.87b	7.20±0.84d	85.00±1.87ab	19.40±1.14cd	84.80±2.59bc	30.20±1.10d	86.00±1.58b	36.40±1.95d
	0.1	23.80±2.05bc	84.00±3.32a	13.00±2.24c	87.00±0.71a	23.80±0.84c	87.20±1.64abc	31.00±1.87c	89.00±1.58a	39.40±2.61c

	0.5	30.40±3.44b	84.80±2.86a	14.80±0.84a	86.20±1.92a	38.40±1.67a	90.00±2.74ab	54.60±1.82a	90.60±1.52a	54.20±2.285a
	1	38.00±3.08a	83.00±1.22a	8.20±0.84b	86.00±2.00a	27.20±3.11b	89.20±2.28ab	40.60±2.88b	89.80±1.64a	51.00±2.12b
	CK	24.80±3.42cd	55.20±10.08c	5.60±1.14d	62.00±5.39c	18.60±1.95cd	65.80±4.97c	28.40±2.07d	76.20±2.17c	36.40±2.3d
Fenofibric acid	0.01	22.80±3.00c	49.60±1.95e	5.40±0.55c	56.00±2.45e	19.80±1.30cd	68.60±1.67d	27.80±1.64e	82.89±2.68c	31.20±2.28c
	0.05	26.20±1.64bc	58.60±4.72bc	8.00±1.00b	60.20±2.28de	22.20±2.49c	72.80±2.77c	30.20±1.64cd	85.00±2.74bc	34.60±2.70b
	0.1	24.20±1.79bc	57.80±4.87bc	5.80±0.45c	67.00±4.74b	22.00±2.34c	75.80±2.39c	32.00±1.22c	84.80±3.19bc	36.00±2.74b
	0.5	32.20±1.30a	63.00±2.65b	7.60±0.89b	76.60±1.82b	25.80±2.28b	84.80±1.48b	36.60±1.52b	87.60±1.95b	37.60±0.89b
	1	33.40±1.95a	74.40±2.07a	10.60±1.14a	84.40±1.140a	28.60±1.87a	90.40±2.30a	43.80±2.17a	91.20±2.28a	48.40±1.52a
	CK	24.80±3.42bc	55.20±10.08cd	5.60±1.14c	62.50±3.85d	18.60±1.95d	65.80±4.97d	28.40±2.07de	76.20±2.17d	36.40±2.30b
Cholini bitartras	0.01	23.00±2.24b	58.00±1.22a	7.80±1.30a	73.00±2.00a	16.00±1.00c	83.00±2.00a	24.80±0.84d	83.40±3.36a	31.80±1.79d
	0.05	13.80±1.64d	53.80±1.10a	6.00±0.71b	73.40±2.51a	21.60±1.14b	81.20±1.10a	31.60±1.82b	82.60±1.51a	39.20±1.30b
	0.1	28.40±2.51a	56.80±2.49a	5.40±1.14b	74.40±2.79a	25.40±1.95a	80.20±2.17a	35.20±1.48a	80.20±1.87a	41.60±0.89a
	0.5	17.60±1.52c	41.40±1.82b	6.20±0.84b	53.20±2.59c	24.60±1.82a	64.00±2.23b	29.00±1.22c	75.00±1.87b	31.60±1.95d
	1	10.20±0.84e	30.60±2.51c	3.20±1.79c	47.40±1.34d	20.40±1.82bc	56.60±1.67c	27.60±1.95c	74.60±1.52b	30.80±1.10d
	CK	24.80±3.42b	55.2±10.08a	5.60±1.14b	62.00±5.39b	18.60±1.95c	65.80±4.97b	28.40±2.07c	76.20±2.17b	36.40±2.30c

2,6-Dihydroxybenzoic acid	0.01	23.40±1.52a	54.60±2.887a	4.40±1.14b	60.20±1.48ab	18.40±2.61a	62.00±1.87b	26.40±1.95a	73.60±2.6a	33.40±1.51b
	0.1	20.00±2.00b	47.60a±3.13b	3.20±0.84c	57.80±3.63b	18.40±1.95a	57.60±2.70c	26.20±2.28a	73.80±2.39a	31.60±1.82b
	0.5	0.00±0.00c	30.80±4.66c	2.80±1.10c	45.60±3.29c	13.40±1.67b	53.00±1.87d	21.60±1.82b	60.20±2.18b	22.20±1.79c
	0.8	0.00c	0.00d	0.00d	1.80±0.84d	0.00c	7.40±1.52e	0.00c	14.40±1.34c	0.00d
	1	0.00c	0.00d	0.00d	0d	0.00c	0.00f	0.00c	0d	0.00d
	CK	24.80±3.42a	55.2±10.08a	5.60±1.14a	62.00±5.39a	18.60±1.95a	65.80±4.97a	28.40±2.07a	76.20±2.17a	36.40±2.30a
Glycyl-L-phenylalanine	0.01	18.20±0.45b	50.80±1.10b	5.60±0.89b	73.40±1.52b	14.60±2.19d	83.60±3.13b	30.60±1.82c	85.80±1.64b	34.60±2.07d
	0.05	19.60±0.89b	53.20±1.48b	6.00±0.71b	69.80±4.76bc	19.80±1.48c	87.40±1.52bc	34.20±1.30b	85.40±2.70b	35.80±1.79cd
	0.1	19.20±3.56b	52.80±2.39b	6.00±1.00b	76.80±1.48b	23.80±1.30b	84.80±2.05b	33.40±2.61b	89.80±4.32a	38.00±2.00c
	0.5	26.60±2.41a	53.60±3.29b	6.60±0.89ab	82.80±3.03a	23.60±2.79b	90.40±2.07ab	34.80±1.48b	89.60±2.61a	47.40±0.55b
	1	25.40±2.07a	65.00±2.74a	7.60±0.89a	85.80±1.79a	28.60±2.70a	92.00±2.45a	46.60±1.52a	92.20±2.17a	57.80±0.45a
	CK	24.80±3.42a	55.20±10.08b	5.60±1.14b	62.00±5.39d	18.60 1.9 c	65.80±4.97d	28.40±2.07c	76.20±2.17c	36.40±2.30cd
Miltefosine	0.01	18.60±2.88b	43.60±2.41b	2.80±1.48b	74.60±1.67a	20.20±1.79a	84.40±2.19a	33.60±2.51a	87.80±1.64a	46.20±2.17a
	0.02	0.00c	0.00c	0.00c	0.80±0.84c	0.00c	1.20±0.84c	0.00c	3.80±0.84b	0.00c
	0.04	0.00c	0.00c	0.00c	0.00c	0.00c	0.40±0.55c	0.00c	0.60±0.55c	0.00c

	0.05	0.00c	0.00c	0.00c	0.00c	0.00c	0.00c	0.00c	0.00c	0.00c
	0.1	0.00c	0.00c	0.00c	0.00c	0.00c	0.00c	0.00c	0.00c	0.00c
	CK	24.80±3.42a	55.20±10.08a	5.60±1.14a	62.00±5.39b	18.60±1.95b	65.80±4.97b	28.4±2.07b	76.20±2.17b	36.40±2.30b

Note: Data in the table represent mean ± standard deviation. Different lowercase letters within the same column indicate statistically significant differences ($p \leq 0.05$) in germination rates between treatments at the same germination time, while identical lowercase letters denote non-significant differences ($p > 0.05$).
